# Phenotyping and comparing the immune cell populations of free-ranging Atlantic bottlenose dolphins (*Tursiops truncatus*) and dolphins under human care

**DOI:** 10.1186/s12917-017-0998-3

**Published:** 2017-03-27

**Authors:** Mahyar Nouri-Shirazi, Brittany F. Bible, Menghua Zeng, Saba Tamjidi, Gregory D. Bossart

**Affiliations:** 1 Charles E. Schmidt College of Medicine, Integrated Medical Science Department, Florida Atlantic University, 777 Glades Road, PO Box 3091, Boca Raton, FL 33431 USA; 2 Department of Gastrointestinal Surgery, The First Affiliated Hospital of Chongqing Medical University, Chongqing, 400016 People’s Republic of China; 3 Georgia Aquarium, 225 Baker Street, NW, Atlanta, GA S USA; 4 Division of Comparative Pathology, Miller School of Medicine, University of Miami, PO Box 016960 (R-46), Miami, FL 33101 USA

**Keywords:** Bottlenose dolphin, Flow cytometry, Immunophenotyping, Marine mammal, Peripheral blood cells

## Abstract

**Background:**

Studies suggest that free-ranging bottlenose dolphins exhibit a suppressed immune system because of exposure to contaminants or microorganisms. However, due to a lack of commercially available antibodies specific to marine mammal immune cell surface markers, the research has been indecisive. The purpose of this study was to identify cross-reactive terrestrial-specific antibodies in order to assess the changes in the immune cell populations of dolphins under human care and free-ranging dolphins. The blood and PBMC fraction of blood samples from human care and free-ranging dolphins were characterized by H&E staining of cytospin slides and flow cytometry using a panel of terrestrial-specific antibodies.

**Results:**

In this study, we show that out of 65 terrestrial-specific antibodies tested, 11 were cross-reactive and identified dolphin immune cell populations within their peripheral blood. Using these antibodies, we found significant differences in the absolute number of cells expressing specific markers within their lymphocyte and monocyte fractions. Interestingly, the peripheral blood mononuclear cell profile of free-ranging dolphins retained an additional population of cells that divided them into two groups showing a low (<27%) or high (>56%) percentage of smaller cells resembling granulocytes.

**Conclusions:**

We found that the cross-reactive antibodies not only identified specific changes in the immune cells of free-ranging dolphins, but also opened the possibility to investigate the causal relationship between immunosuppression and mortality seen in free-ranging dolphins.

## Background

In recent years there has been a drastic increase in the number of cetacean strandings and mortalities along the coast of the United States, which the National Marine Fisheries Service (NMFS) has termed unusual mortality events (UMEs). UMEs have been occurring yearly amongst a variety of marine species in different areas of the U.S. However, one of the largest UMEs which began in July 2013 has affected hundreds of bottlenose dolphins along the Atlantic coast of the U.S. [1]. Although it is often very difficult to determine the cause of an UME, NMFS attributes many to biotoxins, ecological factors, infectious diseases, such as *morbillivirus* [2], or human interactions [3]. Interestingly, studies have shown that high trophic level predators, such as dolphins, have very high concentrations of brevetoxin [4] and chemical pollutants [5] in their tissues as a result of consuming lower trophic level fish that also have high concentrations of these contaminants.

Animal and human studies have demonstrated the importance of the immune system for combating infectious diseases and cancer. Indeed, number of reports suggest a correlation between environmental contaminants, immunosuppression, and diseases susceptibility in marine mammals. For example, several studies have found changes in immunological parameters, such as absolute number of eosinophils and lymphocytes [6] and lymphocyte proliferation in dolphins exposed to certain contaminants [7–9] that may adversely lead to cell anergy or autoimmune diseases [7, 8]. Other studies showed an inverse correlation between contaminant levels and immune cells and their function in marine mammals, such as absolute number of lymphocytes, eosinophils, and monocytes [6], lymphocyte proliferation [10–13], phagocytosis [14], and nonspecific [15] and specific [16] immune responses. The fact that the incidence of tumors is also increasing in free-ranging dolphins suggests that tumors can evade immune surveillance due to changes in their immune parameters [17, 18].

In addition to environmental contaminants, the immunosuppressive effects of infectious diseases in dolphins have also been reported in several studies. For example, dolphins infected with lobomycosis, a mycotic skin disease, displayed a suppressed immune system compared to dolphins without a visible infection [19]. Dolphins with antibody titers positive to bacterial infection, *Chlamydiaceae*, showed changes in their innate and adaptive immunological parameters [20]. Dolphins with positive morbilliviral antibody titers had decreased T cell proliferation and absolute number, suggestive of adverse changes in their immune system [21]. Also, the deceased dolphins affected by UMEs on the Atlantic coast of the U.S. and the Gulf of Mexico had high prevalence of morbilliviral antigens, lesions indicative of a *morbillivirus* infection, and secondary infections [22]. Of note, prior to these studies, it was reported that dolphins affected by an UME in the Gulf of Mexico had high levels of numerous immunosuppressive chemicals and toxins in their liver and opportunistic infections [23].

Overall, these studies [6–16, 19–21, 24] shed light on the immunosuppressive effects of contaminants or microorganisms on dolphins’ immune system. In order to better investigate a cause and effect relationship between contaminants, immunosuppression, diseases susceptibility and mortality leading to UMEs, it is essential to characterize, monitor, and evaluate specific changes in their immune cells. Flow cytometry is commonly used method to monitor the immune status and disease progression in humans and experimental laboratory animals. However, this method has not been utilized in dolphins due to lack of commercially available antibodies specific to marine mammal immune cell surface markers. We addressed this limitation by identifying cross-reactive terrestrial-specific antibodies to phenotype the immune cells of dolphins under human care. We then utilized these selected antibodies to monitor the immune status of free-ranging dolphins by comparing their immune cell subsets to those of dolphins under human care.

## Methods

### Staining media and antibodies

Media containing PBS 1X, 2% heat-inactivated FCS and 2 mM EDTA was used to label cells with monoclonal antibodies. Terrestrial monoclonal antibodies were purchased from BD Bioscience (San Jose, CA) and eBioscience (San Diego, CA). Antibodies tested that were not cross-reactive; *Human*-*specific*: CD1a (HI149), CD19 (SJ25C1), CD19 (1D3), CD62L (DREG56), HLA-DR (G46-6 or L243) (eBioscience, CA), CD3 (UCHT1), CD3 (SP34-2), CD3e (SP34), CD3e (APA1/1), CD4 (M-T477), CD4 (L200), CD8 (SK1), CD8 (HIT8a), CD8 (RPA-T8), CD11b (ICRF44), CD15 (HI98), CD19 (HIB19), CD25 (MA251), CD45 (HI30), Ig Kappa Light Chain (polyclonal), HLA-ABC (G46-2.6), and HLA-ABC (DX17) (BD Bioscience, CA). *Mouse*-*specific*: CD4 (GK1.5), CD8a (53-6.7), CD11b (M1/70), IA-IE (M5/114.15.2) (eBioscience, CA), CD3 complex (17A2), CD3e (145-2C11), CD3e (500A2), CD4 (RM4-5), CD45 (30-F11), and CD49b (DX5) (BD Bioscience, CA). *Rat*-*specific*: CD80 (3H5) (BD Bioscience, CA).

Antibodies tested that were cross-reactive but not selected due to low detectability of corresponding markers; *Human*-*specific*: CD11c (3.9) (eBioscience, CA). *Mouse*-*specific*: CD4 (H129.19), CD4 (RM4-4), CD8b (H35-17.2), CD40 (3/23), CD80 (16-10A1), Ly-6G (1A8), H-2Kb (AF6-88.5) (BD Bioscience), CD11c (N418), CD25 (PC61.5), CD62L (MEL-14), B220 (RA3-6B2), and H2Ld-Db (28-14-8) (eBioscience, CA). *Rat*-*specific*: CD3 (G4.18), CD4 (OX-35), CD8a (OX-8), CD86 (24 F), and RT1A (OX-18) (BD Bioscience, CA). *Pig*-*specific*: CD3e (BB23-8E6-8C8), CD4a (74-12-4), and CD8a (76-2-11) (BD Bioscience, CA).

Antibodies tested that were cross-reactive and selected due to high detectability of corresponding markers; *Human*-*specific*: CD4 (RPA-T4), CD14 (61D3), CD56 (MEM188), HLA-DR (LN3) (eBioscience, CA), CD8 (G42-8), Ig Lambda Light Chain (polyclonal) (BD Bioscience, CA), and HLA-ABC (W6/32) (DAKO). *Mouse*-*specific*: CD40 (HM40-3) and Ly-6G and Ly-6C (RB6-8C) (BD Bioscience, CA). *Rat*-*specific*: CD3 (1F4) and CD11b (WT.5) (BD Bioscience).

Isotype controls for selected cross-reactive antibodies: IgM kappa (G155-228), IgG2a kappa (G155-178), IgA kappa (M18-254), IgM lambda (G235-1), IgG2b (A95-1) (BD Bioscience, CA), and IgG1 kappa (P3.6.2.8.1), IgG2a kappa (eBM2a), and IgG2b kappa (eBMG2b) (eBioscience, CA). Isotype controls for clone HM40-3 and Ig Lambda Light Chain were not available.

### Source of dolphin blood samples

Atlantic bottlenose dolphin (*Tursiops truncatus*) blood samples were obtained from nine captive dolphins under human care at the Georgia Aquarium in Atlanta, Georgia and 16 free-ranging dolphins in the estuarine areas of Charleston, South Carolina as part of the Dolphin Health and Environmental Risk Assessment (HERA) Project. Techniques and protocols related to the dolphin capture-release practices of the HERA Project have been previously described [25]. All samples were shipped overnight and processed immediately. The Atlantic bottlenose dolphin HERA Project was initiated as a multidisciplinary, integrated, collaborative effort in 2003 to assess individual and population health in two southeast coastal regions of the USA: Charleston, SC and the Indian River Lagoon, FL [26]. All methods used in the HERA Project for capture and sample collection were approved under NMFs Scientific Research Permit Nos. 9981678 and 14352-02 issued to G. Bossart and Florida Atlantic University IACUC Protocol Number A10-13. Samples from animals under human care were obtained from the Georgia Aquarium facilities as part of a routine preventative medicine program and were from individual animals with normal clinical history and physical examination.

### Blood processing

Ficoll-Hypaque technique is commonly utilized in humans to isolate PBMCs from peripheral blood to study innate and adaptive immune cells and to use monocytes to generate ex vivo dendritic cells (DCs). We used this method to isolate peripheral blood mononuclear cells (PBMCs) from dolphin peripheral blood by centrifugation of blood samples with Ficoll-Paque PLUS (Amersham Biosciences, NJ) in 50 ml Falcon-tubes for 30 min at 900 × g at 20 °C. The viability of PBMCs was quantitated by trypan blue staining and was >95%. A fraction of the PBMCs were used for labeling with corresponding monoclonal antibodies for further analysis on flow cytometry. Remaining PBMCs were frozen in freezing media containing 90% fetal calf serum (FCS) and 10% dimethyl sulfoxide (DMSO) for further analysis. There were no differences in expression levels of cellular markers observed whether fresh or frozen PBMCs were used for phenotyping.

### Immunophenotyping of PBMCs

Flow cytometry is commonly used to simultaneously measure and analyze multiple characteristics of cells within PBMCs, including size, granularity, and surface marker expression. Prior to labelling, fresh or frozen PBMCs were washed three times in a staining media by resuspending and repeating the centrifugation steps at 600 × g for 5 min. For labeling, cells pre-incubated with mouse and rat serum were stained with the fluorochrome-conjugated antibodies or matching isotype control antibodies for 30 min on ice in staining media. To remove any unbound antibodies, the cells were washed two times by centrifugation at 600 × g for 5 min at 4 °C. Finally, the cells were transferred to flow cytometry sample storage tubes containing staining media and 2% paraformaldehyde and kept at 4 °C before acquisition on FACSCalibur flow cytometer using Cell QuestPro acquisition software (BD Bioscience, San Jose, CA). The data were analyzed using FlowJo software (TreeStar, Ashland, OR).

### Blood smear and cytopsin slide preparations

Blood smears were prepared using peripheral blood from dolphins under human care. Cytospin slides were prepared by spinning isolated PBMCs from dolphins under human care and free-ranging dolphins at 800 rpm for 5 min on a cytospin centrifuge (Shandon Inc., Pittsburgh, PA). Blood smear and cytospin slides were fixed and stained with Fisher Scientific three step stain set. Slides were photographed on an Olympus Provis AX microscope using Olympus cellSens software.

### Statistical analysis

Values are presented as means ± SEM. To determine the significance of data, statistical comparisons were performed using a *t*-test assuming unequal variances. A *p* value <0.05 was statistically significant and shown as an asterisk (*).

## Results

### PBMCs isolated from peripheral blood display differences between dolphins under human care and free-ranging dolphins

**Fig. 1:**
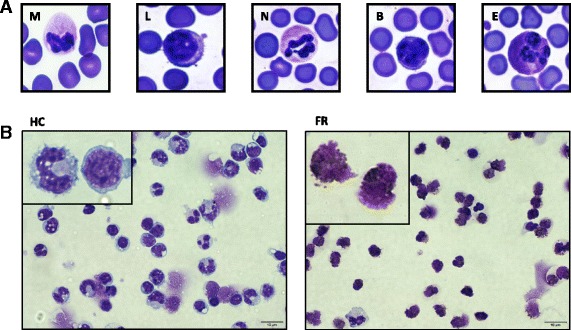
Morphology of peripheral white blood cells. **a** Whole peripheral blood smears of dolphins under human care. M-Monocyte, L-Lymphocyte N-Neutrophil, B-Basophil, and E-Eosinophil. **b** Dolphins under human care (HC) and free-ranging dolphin (FR) isolated peripheral blood mononuclear cells (PBMCs) spun on a cytospin centrifuge (Shandon Inc., Pittsburgh, PA) at 800 rpm for 5 mins. Blood smear and cytospin slides were fixed and stained with Fisher Scientific three step stain set and photographed at 100X and 60X magnification, respectively

Similar to humans and other species, dolphin peripheral blood smears showed a heterogeneous population of white bloods cells (WBCs): monocytes, lymphocytes, and granulocytes, including neutrophils, basophils and eosinophils (Fig. 1a). Using Ficoll-Hypaque technique, we isolated on average 1.3 × 10^6^ PBMCs/ml ranging from 0.9 to 1.5 × 10^6^ PBMCs/ml from peripheral blood of dolphins under human care (Table 1). However, the absolute number of PBMCs isolated from peripheral blood of free-ranging dolphins was significantly higher (three-fold) with an average of 3.1 × 10^6^ PBMCs/ml ranging from 1.3 to 9.0 × 10^6^ PBMCs/ml (Table 1). In addition, cytospin slides of isolated PBMCs from free-ranging dolphins showed differences in cellular composition when compared to dolphins under human care, specifically with a significant increase in granulocytes and decrease in monocytes and lymphocytes (Fig. 1b).

**Table 1:** Absolute number of peripheral blood mononuclear cells (PBMCs)

Dolphin #	Blood Sample Volume (ml)		PBMCs (x10^6^)		PBMCs/ml (x10^6^)		Monocytes (%)		Lymphocytes (%)		Granulocytes (%)	
	HC	FR	HC	FR	HC	FR	HC	FR	HC	FR	HC	FR
1 (A)	16	12	20.6	25	1.3	2.1	17.5	20.8	64.5	41.4	2.74	22.1
2 (A)	14	20	21.8	93	1.5	4.7	24	12	54	58	4.7	9
3 (A)	15	20	18.6	50	1.5	2.5	13.7	14	31	62	15.6	3
4 (A)	16	15	16.8	20	1.4	1.3	12.5	15.3	66	54.9	2.86	13.4
5 (A)	12	20	17.4	74	1.2	3.7	19.4	11	61.5	64	1.78	5
6 (A)	14	20	12.9	86.5	1	4.3	12	6	72	64	1.35	15
7 (A)	11	20	15.6	75.6	0.9	3.8	12	11	67	54	3.02	11.7
8 (A)	15	20	19.1	37.6	1.3	1.9	14	16.6	66	45.5	1.92	27.3
9 (A)	13	20	16.3	92.4	1.2	4.6	28	7.5	48	47	2.27	24
10 (A)		18		30.3		1.7		17.8		49.3		6.9
11 (A)		15		47.4		3.2		7		60.5		16
12 (A)		20		27		1.4		13.5		68.6		6.7
13 (B)		20		32		1.6		7.7		17		56
14 (B)		20		25.4		1.3		4.8		16		58
15 (B)		20		180		9		1.8		9.5		74.5
16 (B)		20		42		2.1		17.6		20		64
Average	14	18.8	17.7	58.6	1.3	3.1	17	11.5	58.9	45.7	4.3	25.8

This table shows the Ficoll-Hypaque gradient isolated PBMC counts. Dolphins under human care dolphins (HC) from Georgia Aquarium (Atlanta, GA) and free-ranging dolphins (FR) from the Dolphin Health and Environmental Risk Assessment (HERA) Project (Charleston, SC). The first column shows the number of dolphins sampled, dolphins under human care (*n* = 9) and free-ranging (*n* = 12 group A and *n* = 4 group B). The second column shows the total volume of blood collected from each dolphin. The third column shows the total number of PBMCs isolated per blood sample. The fourth column shows the total number of PBMCs isolated per ml of blood. The fifth, sixth, and seventh columns show the percentages of monocyte, lymphocyte, and granulocyte fractions within the isolated PBMCs, respectively. Also the table shows the average for each column

### Flow cytometry profile of PBMCs display differences between dolphins under human care and free-ranging dolphins

**Fig. 2:**
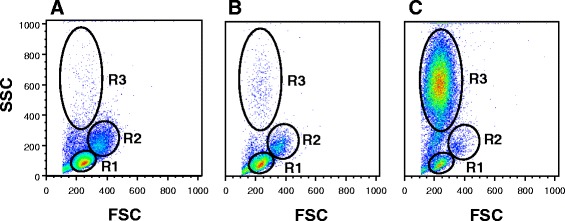
Flow cytometry profile of total PBMCs. The forward-scatter and side-scatter (FSC/SSC) plots represent the total PBMCs profile and lymphocyte (R1), monocyte (R2), and granulocyte (R3) regions. **a** Dolphins under human care lymphocyte, monocyte, and granulocyte regions; **b** group A free-ranging dolphin lymphocyte, monocyte, and granulocyte regions; and **c** group B free-ranging dolphin lymphocyte, monocyte, and granulocyte regions

Similar to human PBMCs, the forward-scatter and side-scatter (FSC/SSC) profile of total isolated PBMCs of dolphins under human care showed two regions, resembling the lymphocyte and monocyte fractions (Fig. 2a). Interestingly, within the FSC/SSC profile of free-ranging dolphins, we observed a third region ranging from low (<27%, group A free-ranging dolphins) to high (>56%, group B free-ranging dolphins) percentage of cells with smaller size and similar granularity but not identical density to granulocytes (Fig. 2b and 2c).

### Terrestrial-specific antibodies identify immune cell subsets within the PBMCs of dolphins

**Fig. 3:**
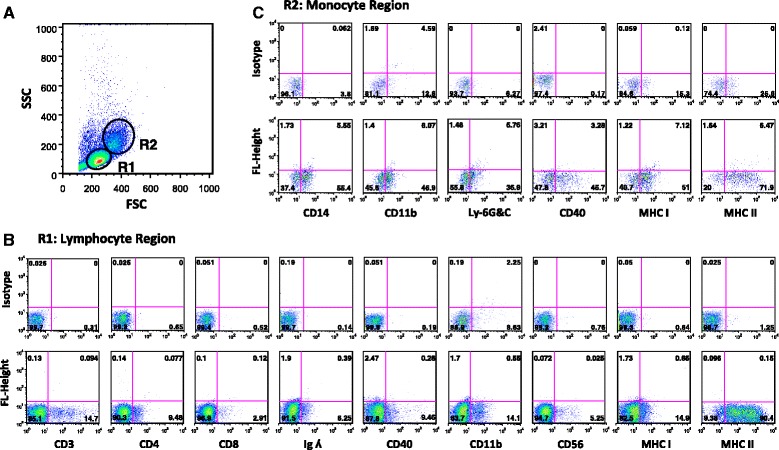
Phenotype of cells within the PBMC gated regions. **a** Shows FSC/SSC profile of dolphins under human care PBMCs gated on lymphocyte and monocyte regions. **b** Represents region 1 lymphocyte fraction with cells expressing the indicated surface markers: CD3/4/8 (T cells), Ig Lambda (λ)/CD40/11b (B cells), CD11b/56 (NK cells), and MHC class I/MHC class II (antigen-presenting cells). **c** Represents region 2 monocyte fraction with cells expressing the indicated surface markers: CD14/11b/Ly-6G&C/40 (monocytes), CD11b/Ly-6G&C (myeloid cells), and MHC class I/MHC class II (antigen-presenting cells). *Quadrants* show the percentage of cells expressing the indicated markers. *Upper plots* represent cells stained with isotype control antibodies

**Fig. 4:**
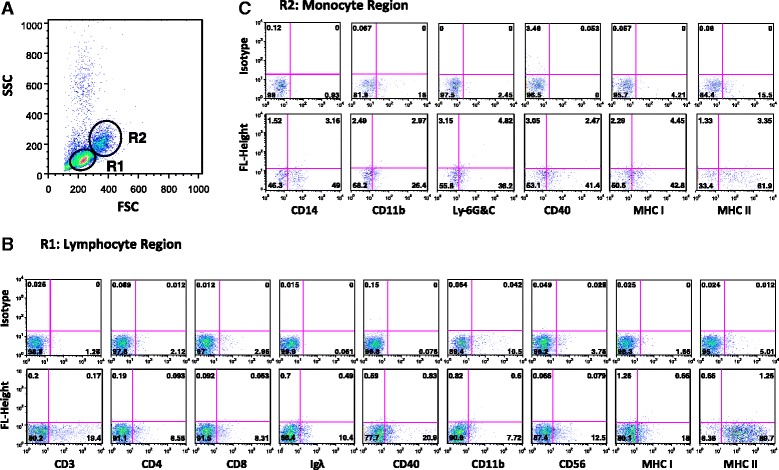
Phenotype of cells within the PBMC gated regions. **a** Shows FSC/SSC profile of group A free-ranging dolphin PBMCs gated on lymphocyte and monocyte regions. **b** Represents region 1 lymphocyte fraction with cells expressing the indicated surface markers: CD3/4/8 (T cells), Ig Lambda (λ)/CD40/11b (B cells), CD11b/56 (NK cells), and MHC class I/MHC class II (antigen-presenting cells). **c** Represents region 2 monocyte fraction with cells expressing the indicated surface markers: CD14/11b/Ly-6G&C/40 (monocytes), CD11b/Ly-6G&C (myeloid cells), and MHC class I/MHC class II (antigen-presenting cells). *Quadrants* show the percentage of cells expressing the indicated markers. *Upper plots* represent cells stained with isotype control antibodies

**Fig. 5:**
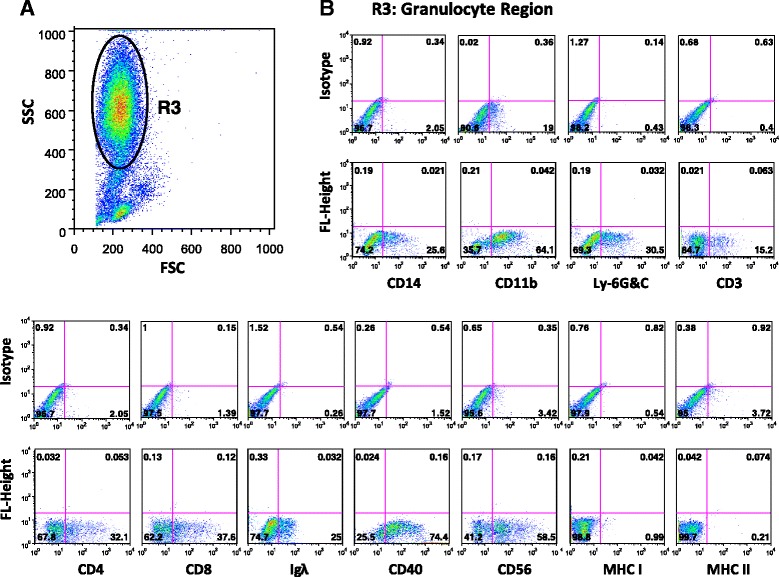
Phenotype of cells within the PBMC gated regions. **a** Shows FSC/SSC of group B free-ranging dolphin PBMCs gated on granulocyte region. **b** Represents region 3 granulocyte fraction with cells expressing the indicated surface markers: CD14/11b/Ly-6G&C/3/4/8/Ig Lambda (λ)/40/56/MHC class I/MHC class II. *Quadrants* show the percentage of cells expressing these indicated markers. *Upper plots* represent cells stained with isotype control antibodies

There are currently no commercially available marine-specific antibodies to distinguish cell types within PBMCs of dolphins. In other species when specific monoclonal antibodies are available lymphocyte fraction (region 1) contains cells that express lymphocyte markers, including T cells (CD3/4/8), B cells (CD19), and NK cells (CD56/49b), while monocyte fractions (region 2) contains cells that express monocyte (CD14/11b) and myeloid markers (CD11b/40), all of them also differentially express major histocompatibility (MHC) antigens (MHC class I/II). In order to identify these immune cells within each region of dolphins’ PBMCs, we took advantage of terrestrial antibodies specific to human, mouse and rat. Amongst the 65 antibodies we tested, 32 showed cross-reactivity of which 11 with the highest cross-reactivity were selected for further identification of these cells within the total PBMCs (Table 2). We found that CD3/4/8, Ig Lambda (λ)/CD40/11b, and CD56/11b monoclonal antibodies were able to distinguish the cells within region 1 as T, B, and NK cells (Fig. 3b) while CD14/11b/Ly-6G&C/40 and CD11b/Ly-6G&C identified monocytes and myeloid cells respectively within region 2 of dolphins under human care (Fig. 3c). In addition, both regions contain cells expressing antigen-presenting molecules, MHC class I and II (Fig. 3b and 3c). These selected monoclonal antibodies were also able to identify the same cell subsets within regions 1 and 2 of normal free-ranging dolphin PBMCs (Fig. 4b and 4c). Interestingly these antibodies detected cells within region 3 of free-ranging dolphins with characteristics of lymphocytes, monocytes, myeloid cells, and granulocytes but lacked expression of antigen-presenting molecules MHC class I and II (Fig. 5b).

**Table 2:** Antibodies tested on dolphin Peripheral Blood Mononuclear Cells (PBMCs)

Antibody	Clone	Species	Antibody	Clone	Species
Not cross-reactive			Cross-reactive, not selected		
CD1a	HI149	Human^a^	CD3	G4.18	Rat^b^
CD3	17A2	Mouse^b^	CD3e	BB23-8E6-8C8	Pig^b^
CD3	UCHT1	Human^b^	CD4	H129.19	Mouse^b^
CD3	SP34-2	Human^b^	CD4	RM4-4	Mouse^b^
CD3e	SP34	Human^b^	CD4	OX-35	Rat^b^
CD3e	APA1/1	Human^b^	CD4a	74-12-4	Pig^b^
CD3e	145-2C11	Mouse^b^	CD8a	OX-8	Rat^b^
CD3e	500A2	Mouse^b^	CD8a	76-2-11	Pig^b^
CD4	M-T477	Human^b^	CD8b	H35-17.2	Mouse^b^
CD4	L200	Human^b^	CD11c	3.9	Human^a^
CD4	RM4-5	Mouse^b^	CD11c	N418	Mouse^a^
CD4	GK1.5	Mouse^a^	CD25	PC61.5	Mouse^a^
CD8	SK1	Human^b^	CD40	23-Mar	Mouse^b^
CD8	HIT8a	Human^b^	CD62L	MEL-14	Mouse^a^
CD8	RPA-T8	Human^b^	CD80	16-10A1	Mouse^b^
CD8a	53-6.7	Mouse^a^	CD86	24F	Rat^b^
CD11b	M1/70	Mouse^a^	B220	RA3-6B2	Mouse^a^
CD11b	ICRF44	Human^b^	Ly-6G	1A8	Mouse^b^
CD15	HI98	Human^b^	MHC I (RT1A)	OX-18	Rat^b^
CD19	HIB19	Human^b^	MHC I (H-2Kb)	AF6-88.5	Mouse^b^
CD19	SJ25C1	Human^a^	MHC I (H2Ld-Db)	28-14-8	Mouse^a^
CD19	1D3	Mouse^a^	Cross-reactive, selected		
CD25	MA251	Human^b^	CD3	1F4	Rat^b^
CD45	HI30	Human^b^	CD4	RPA-T4	Human^a^
CD45	30-F11	Mouse^b^	CD8	G42-8	Human^b^
CD49b	DX5	Mouse^b^	CD11b	WT.5	Rat^b^
CD62L	DREG56	Human^a^	CD14	61D3	Human^a^
CD80	3H5	Rat^b^	CD40	HM40-3	Mouse^b^
MHC I (HLA-ABC)	G46-2.6	Human^b^	CD56	MEM188	Human^a^
MHC I (HLA-ABC)	DX17	Human^b^	Ly-6G and Ly-6C	RB6-8C5	Mouse^b^
MHC II (HLA-DR)	G46-6 or L243	Human^a^	MHC I (HLA-ABC)	W6/32	Human^c^
MHC II (IA-IE)	M5/114.15.2	Mouse^a^	MHC II (HLA-DR)	LN3	Human^a^
Ig κ Light Chain	Polyclonal	Human^b^	Ig λ Light Chain	Polyclonal	Human^b^

Table lists the terrestrial-specific antibodies that were not cross-reactive, cross-reactive but not selected, or cross-reactive and selected. Letters denote the companies where the antibodies were purchased. ^a^eBioscience, ^b^BD Bioscience, ^c^DAKO

### Frequency of immune cells within the PBMCs of dolphins under human care and free-ranging dolphins expressing specific surface markers

**Fig. 6:**
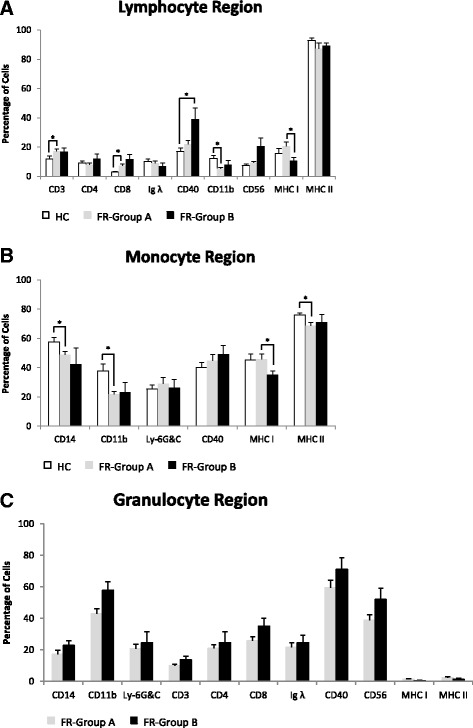
Percentage of cells within lymphocyte, monocyte, and granulocyte regions. **a** Represents the average percentage of cells expressing indicated surface markers within the lymphocyte region of dolphins under human care (HC) and group A (FR-Group A) and B (FR-Group (**b**) free-ranging dolphins. **b** Represents the average percentage of cells expressing indicated surface markers within the monocyte region of dolphins under human care (HC) and group A (FR-Group A) and B (FR-Group B) free-ranging dolphins. **c** Represents the average percentage of cells expressing indicated surface markers within the granulocyte region of group A (FR-Group A) and B (FR-Group B) free-ranging dolphins. Sample sizes are HC (*n* = 9), FR-Group A (*n* = 12), and FR-Group B (*n* = 4). Values are presented as means ± SEM. An asterisk (*) denotes statistical significance

**Fig. 7:**
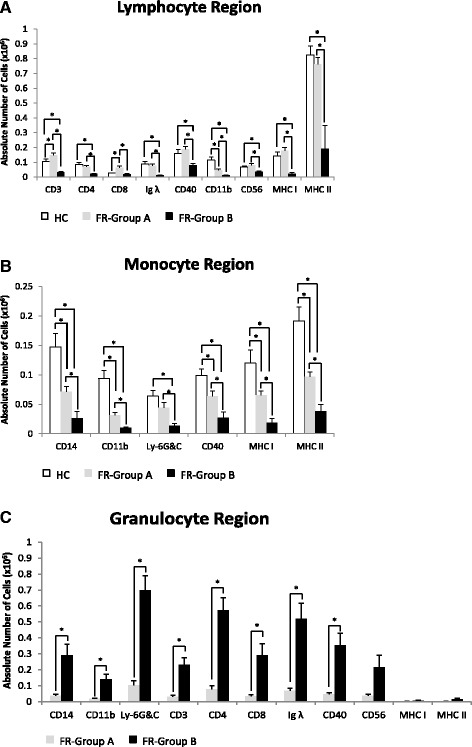
Absolute number of cells within lymphocyte, monocyte, and granulocyte regions. **a** Represents the average absolute number of cells expressing indicated surface markers within the lymphocyte region of dolphins under human care (HC) and group A (FR-Group A) and B (FR-Group B) free-ranging dolphins. **b** Represents the average absolute number of cells expressing indicated surface markers within the monocyte region of dolphins under human care (HC) and group A (FR-Group A) and B (FR-Group B) free-ranging dolphins. **c** Represents the average absolute number of cells expressing indicated surface markers within the granulocyte region of group A (FR-Group A) and B (FR-Group B) free-ranging dolphins. Sample sizes are HC (*n* = 9), FR-Group A (*n* = 12), and FR-Group B (*n* = 4). Values are presented as means ± SEM. An asterisk (*) denotes statistical significance

Our data shows that the differences in FSC/SSC profiles observed between dolphins under human care and free-ranging dolphins reflect increases or decreases in average percentage and absolute number of each immune cell within the lymphocyte, monocyte, and granulocyte regions. Indeed, the average percentage of cells in the lymphocyte region expressing CD4, Ig λ, CD56 and MHC class II surface markers was similar between dolphins under human care and group A and B free-ranging dolphins (Fig. 6a). However the percentage of cells expressing CD3 and CD8 were significantly increased while CD11b was decreased in group A and CD40 was increased in group B free-ranging dolphins when compared to dolphins under human care (Fig. 6a). In addition, we observed a significant increase in percentage of cells expressing MHC class I in group A when compared to group B free-ranging dolphins (Fig. 6a). Then we compared the absolute number of dolphins under human care and free-ranging dolphins’ cells within the lymphocyte region expressing the indicated markers. We found a significant increase in absolute number of group A free-ranging dolphin cells expressing CD3 and CD8 and a decrease in CD11b when compared to dolphins under human care. Finally, group B free-ranging dolphins showed a significant decrease in the absolute number of cells expressing all markers and all markers except CD8 when compared to group A free-ranging dolphins and dolphins under human care, respectively (Fig. 7a). We also compared the differences in the percentage and absolute number of cells within the monocyte region between dolphins under human care and free-ranging dolphins. There was a significant decrease in the percentage of group A free-ranging dolphin cells expressing CD14, CD11b, and MHC class II compared to dolphins under human care (Fig. 6b). There was also a significant decrease in percentage of group B free-ranging dolphin cells expressing MHC class I when compared to group A free-ranging dolphins (Fig. 6b). When the absolute number of cells within the monocyte region was compared, we found a significant decrease in the absolute number of group A free-ranging dolphin cells expressing all markers, except Ly-6G&C when compared to dolphins under human care (Fig. 7b). In addition, we found group B free-ranging dolphins had a significant decrease in absolute number of cells expressing all markers when compared to dolphins under human care and group A free-ranging dolphins (Fig. 7b). Finally, we compared the average percentage and absolute number of cells expressing each indicated marker within the granulocyte region only seen in group A and B free-ranging dolphins. We found that group B with a high increase in granulocytes on FSC/SSC plots showed a greater percentage of cells for almost all surface markers, although not significant (Fig. 6c). Also, the absolute number of cells expressing each indicated marker was significantly increased in group B compared to group A free-ranging dolphins except CD56, MHC class I, and MHC class II (Fig. 7c).

## Discussion

In this study, we investigated dolphins under human care that served as a healthy control model for dolphins subject to routine health care in order to characterize their immune cell subsets and compare them side by side to those of free-ranging dolphins. Using a hemocytometer, we found that Atlantic bottlenose dolphins under human care have on average 6.5 × 10^6^ white blood cells (WBCs)/ml of blood (Table 3) which is lower than 10.95 × 10^6^ WBCs/ml reported in previous studies using an automatic cell counter [27, 28]. This discrepancy is most likely due to differences in counting methods. Indeed, using an automatic cell counter, others [25, 29] and we (Table 3) found that free-ranging Atlantic bottlenose dolphins have 11.0 × 10^6^ WBCs/ml of blood and 12.7 × 10^6^ WBCs/ml, respectively. Within the WBCs, studies showed that dolphins under human care have 2.3% monocytes, 21.7% lymphocytes, 60.3% neutrophils, <1% basophils, and 14.5% eosinophils [27, 28, 30, 31], while free-ranging dolphins have 2.9% monocytes, 21.1% lymphocytes, 40.3% neutrophils, <1% basophils, and 35.9% eosinophils [25, 29]. We observed that dolphins under human care have 3.9% monocytes, 17.8% lymphocytes, 63.3% neutrophils, 1.2% basophils, and 12.1% eosinophils, while free-ranging dolphins have 2.7% monocytes, 17% lymphocytes, 39.5% neutrophils, 0.9% basophils, and 39.6% eosinophils within their WBCs (Table 3). The decrease in lymphocytes and neutrophils and increase in eosinophils that we observed in free-ranging compared to dolphins under human care is consistent with the WBC differentials reported in the previous studies [25, 27–31]. Of note, dolphins under human care and free-ranging dolphins have a leukocyte differential similar to humans, except their eosinophil count which is much higher than the 1–4% range seen in humans.

**Table 3:** Absolute number of white blood cells (WBCs)

Dolphin #	Sex		Age (years)		Health Status		WBCs/ml (x10^6^)		Monocytes (%)		Lymphocytes (%)		Neutrophils (%)		Basophils (%)		Eosinophils (%)	
	HC	FR	HC	FR	HC	FR	HC	FR	HC	FR	HC	FR	HC	FR	HC	FR	HC	FR
1 (A)	M	M	12	31^a^	Normal	Possibly diseased	7.6	11.2	3.3	0.9	14	7.1	74	40.2	1	0	8.3	51.8
2 (A)	M	M	21	32^a^	Normal	Diseased	7	16.2	5.7	1.2	13	17.3	61	32.1	1	0	20	50
3 (A)	F	M	13	26^a^	Normal	Normal	7.6	9.8	2	2	15.7	25.5	65	21.4	2	2	16	50
4 (A)	F	F	8	35^b^	Normal	Normal	4.5	8.1	2	4.9	27	29.6	55	42	1	1.2	15.7	22.2
5 (A)	F	F	11	^e^	Normal	Possible Disease	7.5	11.3	5	1.8	19	17.7	49	32.7	1	1.8	26	45.1
6 (A)	F	F	8	18^a^	Normal	Diseased	5.2	19	2.7	1.1	17	23.2	73	32.1	1.5	3.2	6.3	41.1
7 (A)	M	M	11	^e^	Normal	Possible Disease	4.4	14.9	7	0.7	4	24.8	65	38.9	1	2	4	32.9
8 (A)	M	F	11	^e^	Normal	Possibly diseased	8.7	13.8	2	2.2	29	24.6	61	34.1	1	0.7	7	37.7
9 (A)	F	M	12	8^c^	Normal	Possibly diseased	6.1	13.8	5.3	0.7	21.3	13.8	67	39.1	1	0.7	5.7	44.9
10 (A)		M		33^c^		Diseased		11.5		4.3		4.3		34.8		0		57.4
11 (A)		M		33^a^		Possibly diseased		11.3		4.4		13.3		36.3		0		46
12 (A)		F		^d^		Diseased		8.1		8.6		19.8		42		0		29.6
13 (B)		F		^F^		Diseased		9.9		3		11.1		54.5		0		31.3
14 (B)		M		25^a^		Diseased		13.1		2.3		12.2		58		0.8		26.7
15 (B)		M		17^c^		Diseased		18.7		3.7		11.8		48.7		1.1		33.7
16 (B)		F		9.5^a^		Possibly diseased		12.9		0.8		16.3		45		0.8		32.6
Average							6.5	12.7	3.9	2.7	17.8	17	63.3	39.5	1.2	0.9	12.1	39.6

This table shows the total WBC counts. Dolphins under human care dolphins (HC) from Georgia Aquarium (Atlanta, GA) and free-ranging dolphins (FR) from the Dolphin Health and Environmental Risk Assessment (HERA) Project (Charleston, SC). The first column shows the number of dolphins sampled, dolphins under human care (*n* = 9) and free-ranging (*n* = 12 group A and *n* = 4 group B). The second column shows the sex of each dolphin. The third column shows the age of each dolphin: HC dolphins were born under human care and their exact birthdate is known while free-ranging dolphins’ ages were: ^a^determined from previous capture; ^b^estimated from Photo-ID;^c^estimated from tooth; ^d^classified as adult based on pregnancy; ^e^unknown and classified as juvenile; and ^F^unknown and classified as adult. The fourth column shows the classification of each dolphins’ health status. The fifth column shows the total number of WBCs per ml of blood. The sixth, seventh, eighth, ninth, and tenth columns show the percentages of monocyte, lymphocyte, neutrophil, basophil, and eosinophil fractions within the total WBCs, respectively. Also the table shows the average for each column

The FSC/SSC flow cytometery profile of free-ranging dolphins’ PBMCs revealed that they can be divided into group A and B based on low (<27%) and high (>56%) percentage of granulocytes respectively (Fig. 2b and 2c). In addition, group B free-ranging dolphins have the highest average PBMC count of 3.5 × 10^6^ per ml compared to 2.9 × 10^6^ per ml for group A and 1.3 × 10^6^ per ml for dolphins under human care (Table 1). Furthermore, the PBMCs of group B free-ranging dolphins have on average a differential of 63% granulocytes, 8% monocytes, and 16% lymphocytes while group A has 13% granulocytes, 13% monocytes, and 56% lymphocytes, and dolphins under human care have 17% monocytes and 59% lymphocytes (Table 1). Therefore, our data revealed that group B free-ranging dolphins have the lowest lymphocyte and monocyte fractions of PBMCs compared to dolphins under human care and group A free-ranging dolphins. Consistent with our flow data, cytospin slides prepared from PBMCs of group B free-ranging dolphins also showed a decrease in lymphocytes and monocytes and an increase in granulocytes compared to dolphins under human care (Fig. 1b). Noteworthy, these observed changes between dolphins under human care, group A, and group B free-ranging dolphins were not unique to their PBMCs since we found similar changes in total WBC count and percentage of lymphocytes, monocytes, and granulocytes within their WBCs. Indeed, group B compared to group A free-ranging dolphins on average have an increase in total WBC count 13.7 × 10^6^ vs. 12.4 × 10^6^ per ml and granulocytes 83.8% vs. 78.8% and a decrease in lymphocytes 12.8% vs. 18.5% and monocytes 2.5% vs. 2.8% (Table 3). Interestingly, within the granulocyte fraction of total WBCs, group B free-ranging dolphins also have an increase in neutrophils 51.8% vs. 35.4% and decrease in eosinophils 31.3% vs. 42.4% compared to group A (Table 3).

Previous studies have labelled cells within various dolphin tissues using human-specific antibodies [32, 33], which suggest that human and dolphin cells share epitopes on their cell surface markers. Our data further suggests that cell surface markers on dolphin immune cells share epitopes with humans and other species, since three of three pig, seven of eight rat, 14 of 25 mouse and eight of 29 human specific antibodies were cross-reactive. Interestingly, out of the 11 antibodies we selected to label dolphin immune cells, seven human, two mouse, and two rat-specific antibodies had the highest cross-reactivity. Therefore, our data demonstrated that using pan surface markers commonly used in mouse and human studies, we could detect dolphin immune cell subsets, including CD3 T cells, CD56 NK cells, and CD14/CD11b monocytes (Fig. 3 and 4). However, we were not able to detect B cells in dolphins using the monoclonal antibody specific to pan B cell marker CD19. Thus, we instead utilized Ig lambda (λ) and CD40, as well as CD11b known also to detect B cell subsets (Fig. 3b and 4b). De Guise et al., [34], reported that 14.1% and 22.63% of Atlantic bottlenose dolphins’ PBMCs expressed CD19 and CD21, respectively using non-commercially available antibodies. However, these antibodies were not available to us for comparison. Altogether, our selected antibodies were able to detect dolphin immune cell subsets, even though they may not have optimally represented the true percentage of immune cells and expression of their markers within dolphin peripheral blood.

In humans and rodents, MHC class II expression is restricted to antigen-presenting cells. We observed that nearly all dolphin B and T lymphocytes and monocytes express MHC class II (Fig. 3 and 4). Our finding is consistent with previous studies that showed more than 90% of bottlenose dolphins’ [35] and beluga whales’ [36] peripheral blood cells within lymphocyte region express MHC class II, which is also observed in ungulates and carnivores such as swine [36], equine [37], canine [38], and feline [39]. Although, MHC class I is displayed on all human and mouse nucleated cells, we found that only a small percentage of dolphin cells expressed MHC class I (Fig. 3 and 4). Furthermore, our data revealed that a similar percentage of cells expressed β2-microglobulin, an invariant chain associated with MHC class I alpha chain (data not shown). Using bovine-specific antibodies, De Guise et al. [36], reported that majority of the cells within the lymphocyte region of beluga whales expressed MHC class I. Whether the discrepancies in MHC class I expression in these studies was due to the antibodies used for labelling or represents their true expression patterns needs further investigation.

The WBC profile of dolphins contained two populations of small and large cells with similar granularity within granulocyte region (data not shown). During PBMC isolation, Ficoll removed nearly all of the large but not small granulocyte-like cells from blood samples of dolphins. Interestingly however, the percentage of low density small granulocyte-like cells retained within PBMCs of free-ranging dolphins, which can also be seen on the cytospin slides (Fig. 1b) was significantly higher than those within PBMCs of dolphins under human care (Fig. 2). In addition, these small granulocyte-like cells expressed all of the markers, except MHC class I and II (Fig. 5b). Interestingly, we observed that group B free-ranging dolphins with the highest percentage of granulocytes in their PBMCs were classified as diseased animals (Table 3). Whether the observed changes in percentage, absolute number, and surface markers of cells within PBMCs of dolphins under human care and group A and B free-ranging dolphins was due to increase in precursor cells, differences in maturational stages of cells or dolphins’ health status merits further evaluation.

## Conclusions

Alterations in WBC count and distribution could provide insight into the health status of dolphins, Indeed, It was found that a bottlenose dolphin under human care infected with Aspergillus fumigatus had a higher than normal WBC count which decreased after treatment [40]. Another study found that seals fed fish contaminated with immunosuppressive organochlorines had an increase in granulocytes which the authors stated may indicate bacterial infections [13]. However, alterations in WBC count offers little information on specific changes imposed by environmental factors on immune cell subsets and their function. In this study, we have identified cross-reactive terrestrial-specific antibodies that allowed us to characterize the immune cell subsets of dolphins under human care and free-ranging dolphins. Our findings opened the possibility of utilizing flow cytometry for routine health assessment by monitoring specific changes in immune cells of dolphins caused by environmental contaminants or infectious agents with the goal of understanding the pathogenesis of diseases.

## Abbreviations

DC: Dendritic cells
FACS: Fluorescence activated cell sorting
FR: Free-ranging dolphin
FSC/SSC: Forward scatter/side scatter
H&E: Hematoxylin and eosin
HC: Human care
HERA: Health and Environmental Risk Assessment
IACUC: Institutional Animal Care Unit Committee
MHC: Major histocompatibility complex
NK: Natural killer cells
NMFS: National Marine Fisheries Service
PBMC: Peripheral blood mononuclear cells
UMEs: Unusual mortality events
WBCs: White bloods cells
